# Inhalation of Conium and Iodine in Tubercular Phthisis Pulmonalis

**Published:** 1841-11

**Authors:** 


*Inhalation of Conium and Iodine in Tubercular Phthisis Pulmonalis.*—Sir Charles Scudamore, in a paper in the *London Med. Gazette*, Feb. 7, 1840, states that he has tried the following method of inhalation in phthisis and bronchitis, sufficiently to enable him to recommend it very strongly to the profession. He denies that the inhalation of conium and iodine in either of the above named diseases irritates the lungs; on the other hand, he says that the patient looks forward with pleasure to the time when the process is to be repeated. He disagrees with Laennec in some points, and thinks that the *early* stages of phthisis may be cured by this mode of treatment, and goes on to relate cases where a cavity was clearly indicated, and where pectoriloquism was most evident, and yet the patient recovered by means of inhalation. Indeed some of his cases were so clearly cases of incipient phthisis, according to his description, that we cannot help placing dependence upon his treatment in some measure, however contrary to the common feelings of the profession. Some, indeed, relapsed and eventually died after an interval of some years; others have not hitherto relapsed, and are yet in perfect health. The author then goes on to relate his method, and the ingredients of his mixture, combining, however, at the same time, other treatment of a general kind. He says—I cannot, I think, too often repeat, that while I claim for the inhalation so great a regard, I consider it to be only one part of the treatment required. The additional constitutional means embrace a very wide consideration. The local external treatment of the chest by proper means of counter-irritation, and by lotions and frictions, is a very important part of management.

I have never seen, from an active remedy, so large a proportion of benefit, with so small a proportion of disagreement and inconvenience, as from the inhalation of iodine and conium. The method also is to be considered; and I may here remark that many excellent remedies have fallen into odium and neglect, at different periods, from the error or abuse of their application. I am careful that all the ingredients which enter into the composition of the inhaling mixture are perfectly pure. I recommend the following formula. ℞.—Iodinii puri; iodid potassii, aa gr. vi; aquæ distillat. ℥v, ℥vi; alcoholis ʒii. M. fiat mistura, in inhalationem adhibenda.

I now always prefer to add the conium at the time of mixing the iodine solution with the water; and it should be a *saturated* tincture, prepared with the most genuine dried leaves. In the commencement of the treatment, I advise very small proportions of the iodine mixture; for example, only from half a drachm to a drachm for an inhaling of eight or ten minutes, to be repeated two or three times a day. Of the soothing tincture, I direct half a drachm—which I usually find sufficient; but it may be increased if the cough be very troublesome. I soon augment the quantity of the iodine, and progressively from ʒi to ʒiv; but also, then prolonging the time of inhaling, I divide the iodine dose, putting two thirds at first, and the rest after the expiration of seven or eight minutes. If the temperature of the water be measured by the thermometer, it should be 120° Fahr. as being the most favorable for volatilizing the active principles of the iodine and conium, mixed with some watery vapour; but the approximation will be sufficient, if equal parts of boiling and cold water be used; with which the inhaler is not to be quite half filled. Invariably, however, care should be taken to prepare the bottle for this heat of water, by first washing it out with some tepid water.

During the process, the inhaler should be kept immersed in a jug, containing water of rather higher temperature than 120°.

It is of the utmost importance that the strength of the inhaling mixture should be considered in relation to the particular case. The feelings of the patient will be a great guidance; he should have the sense of relief, and not of inconvenient irritation, produced. The cough arising occasionally during the process is not an objection; but if it be more irritable afterwards, it shows that it has been used too strong. There is a certain stage of the tubercular disease, when over-excitement, from employing the iodine in too great quantity, might hurry on the softening process too quickly. It is here that the treatment demands the greatest judgment. In every case one of the following events may be expected to happen; either that the tubercular irritation will be arrested and gradually removed, be arrested and suspended, but not cured, or pass on to the softening process, which terminates in the production of an excavation. In all these different states of disease, I advise the inhaling treatment to be employed.

It is my belief that this direct and very accurate mode of applying this powerful medicine, iodine with conium, induces a new action in the vessels and nerves of the lungs, which is calculated to supercede the diseased action. I also assign much effect to the stimulation of the absorbents, and have been led to believe that tubercles have in this manner been actually removed.

With a well constructed glass inhaler I find all the satisfaction I can desire. The bottle should be large, and the tubes capacious. The one issuing from the bottles should be upright, passing off in a gradual slight curve, so that the vapour shall not be much cooled in the course of its progress; the ingress tube should dip very near to the bottom of the bottle, that all the air so introduced may receive impregnation. The patient must be desired to inhale by using at the same time suction and a pretty full inspiration, then to drop the under lip from the mouth-piece and make a free expiration; so conducting the process by pausing, and, if he like, little suspensions, in order that he may not experience any of the fatigue which would certainly happen if breathing quickly, or using an inhaler with small tubes, or with too much water in the bottle.—*Braithwaite's Retrospect*, from *Lond. Med. Gaz.*, Feb. 1840; *Ibid.*

